# Prolonged inhibition of 5-HT_3_ receptors by palonosetron results from surface receptor inhibition rather than inducing receptor internalization

**DOI:** 10.1111/bph.12204

**Published:** 2013-06-21

**Authors:** J Daniel Hothersall, Christopher Moffat, Christopher N Connolly

**Affiliations:** Division of Neuroscience, Medical Research Institute, Ninewells Medical School, University of DundeeDundee, UK

**Keywords:** 5-HT_3_ receptor, palonosetron, emesis, cancer therapy, irreversible antagonism, endocytosis, down-regulation, receptor trafficking, allosterism

## Abstract

**Background and Purpose:**

The 5-HT_3_ receptor antagonist palonosetron is an important treatment for emesis and nausea during cancer therapy. Its clinical efficacy may result from its unique binding and clearance characteristics and receptor down-regulation mechanisms. We investigated the mechanisms by which palonosetron exerts its long-term inhibition of 5-HT_3_ receptors for a better understanding of its clinical efficacy.

**Experimental Approach:**

Cell surface receptors (recombinantly expressed 5HT_3_A or 5HT_3_AB in COS-7 cells) were monitored using [^3^H]granisetron binding and ELISA after exposure to palonosetron. Receptor endocytosis was investigated using immunofluorescence microscopy.

**Key Results:**

Chronic exposure to palonosetron reduced the number of available cell surface [^3^H]granisetron binding sites. This down-regulation was not sensitive to either low temperature or pharmacological inhibitors of endocytosis (dynasore or nystatin) suggesting that internalization did not play a role. This was corroborated by our observation that there was no change in cell surface 5-HT_3_ receptor levels or increase in endocytic rate. Palonosetron exhibited slow dissociation from the receptor over many hours, with a significant proportion of binding sites being occupied for at least 4 days. Furthermore, our observations suggest that chronic receptor down-regulation involved interactions with an allosteric binding site.

**Conclusions and Implications:**

Palonosetron acts as a pseudo-irreversible antagonist causing prolonged inhibition of 5-HT_3_ receptors due to its very slow dissociation. In addition, an irreversible binding mode persists for at least 4 days. Allosteric receptor interactions appear to play a role in this phenomenon.

## Introduction

The 5-HT_3_ receptor is a cationic ligand-gated ion channel that mediates neuronal depolarization in both the central and peripheral nervous system in response to 5-HT (receptor nomenclature follows Alexander *et al*., 2011). The 5-HT_3_ receptors belong to the Cys-loop superfamily of ligand-gated ion channels that includes receptors for acetylcholine, GABA and glycine (Connolly and Wafford, 2004). In humans, there are five 5-HT_3_ receptor subunits (5-HT3A-E) (Walstab *et al*., 2010; Kapeller *et al*., 2011) with homomeric 5-HT_3_A and heteromeric 5-HT_3_AB receptors being the most common and best characterized (Barnes *et al*., 2009). The 5-HT_3_ receptors are found in many regions of the CNS including the hippocampus, entorhinal cortex and frontal cortex (Faerber *et al*., 2007), and also play an important role in the enteric nervous system (Hansen and Witte, 2008).

The 5-HT_3_ receptor antagonists are important tools in cancer therapy to control emesis and nausea during chemotherapy, radiotherapy, surgery or anaesthesia (Aapro, 2007; Muchatuta and Paech, 2009). Emesis is thought to involve 5-HT release from enterochromaffin cells in the intestinal mucosa, causing activation of 5-HT_3_ receptors on abdominal vagal afferents that stimulate the emetic reflex (Hornby, 2001; Minami *et al*., 2003). The antiemetic activity of 5-HT_3_ receptor antagonists is believed to involve inhibition of these peripheral receptors, although a role of 5-HT_3_ receptor antagonism in the CNS has also been proposed (Costall and Naylor, 1992; Hornby, 2001; Minami *et al*., 2003).

Numerous clinical trials have indicated that palonosetron (Aloxi®; RS 25259 197) is more effective than other 5-HT_3_ receptor antagonists in the treatment of emesis, and is particularly useful in the setting of delayed emesis and in reducing nausea, where other drugs targeting 5-HT_3_ receptors lack efficacy (Aapro, 2007; Rojas *et al*., 2008; Muchatuta and Paech, 2009; Rojas and Slusher, 2012; Schwartzberg *et al*., 2012). The unique pharmacological and pharmacokinetic properties of palonosetron have been proposed as explanations for these findings. Importantly, palonosetron has a longer plasma half-life (Eisenberg *et al*., 2004; Stoltz *et al*., 2004) and displays additional allosteric interactions with the 5-HT_3_ receptor (Rojas *et al*., 2008; Moura Barbosa *et al*., 2010). Palonosetron has also been proposed to cause prolonged inhibition of 5-HT_3_ receptors by promoting receptor internalization (Rojas *et al*., 2010b). Additionally, palonosetron's ability to interfere with the crosstalk between the 5-HT_3_ receptor and the neurokinin-1 receptor, another important target for antiemetic therapy, might be involved in its prolonged efficacy (Rojas *et al*., 2010a).

Little is known regarding the mechanisms that govern the regulation of cell surface 5-HT_3_ receptor expression, and hence receptor function. Constitutive internalization of 5-HT_3_A receptors has been observed in recombinant systems (Ilegems *et al*., 2004; Morton *et al*., 2011) and expression of brain-derived neurotrophic factor attenuates the 5-HT_3_ receptor expression *in vivo* (Huang and Morozov, 2011) by unknown mechanisms. In terms of exocytosis, it has been reported that 5-HT_3_A receptor assembly and trafficking signals (Boyd *et al*., 2002; 2003) and post-translational modification (Sun *et al*., 2003; Monk *et al*., 2004; Massoura *et al*., 2011) control recruitment to the cell surface. Recently, a novel chaperone molecule, resistance to inhibitors of cholinesterase type 3 (RIC-3), has been shown to differentially promote the surface expression of 5-HT_3_A, but not 5-HT_3_AB (Cheng *et al*., 2005; 2007), or 5-HT_3_AC-E receptors (Walstab *et al*., 2010). Ligand-induced regulation of 5-HT_3_ receptors may promote long-term changes in receptor function that are distinct from acute pharmacological activity. Indeed, 5-HT_3_ receptor agonists and antagonists have been suggested to modulate cell surface receptor levels (Ilegems *et al*., 2004; Freeman *et al*., 2006; Rojas *et al*., 2010b), with receptor internalization being reported in the enteric nervous system following excess 5-HT (Freeman *et al*., 2006) and in recombinant systems, following exposure to palonosetron (Rojas *et al*., 2010b). These findings may be relevant to the clinical efficacy of drugs targeting the receptor, and for pathological circumstances of high concentrations of 5-HT.

The mechanisms by which palonosetron exerts its long-term inhibition of human 5-HT_3_ receptors, while other antagonists provide only short-term actions, could result from its unique receptor binding actions and/or subsequent receptor trafficking. Therefore, we sought to address these issues using cellular approaches. We found that palonosetron induced a long-term down-regulation in the number of available 5-HT_3_ receptor binding sites that persisted for greater than 24 h, and that recovery from this inhibition did not require the delivery of new surface receptors, suggesting that there was instead slow dissociation from the receptor. Furthermore, palonosetron did not reduce the cell surface expression of 5-HT_3_ receptors, and was without effect on rate of receptor endocytosis. We propose that prolonged inhibition of 5-HT_3_ receptors occurred through pseudo-irreversible interactions with the receptor, rather than by promoting internalization. In addition, we also identify an additional binding mode with irreversible (over 96 h) receptor inhibition.

## Methods

### Cell culture and transfection

Simian COS-7 cells (ACC CRL 1651) were maintained in DMEM supplemented with 10% FBS, 2 mM L-glutamine, 1 mM sodium pyruvate, 100 μg·mL^−1^ streptomycin and 100 U·mL^−1^ penicillin in an atmosphere of 5% CO_2_ at 37°C. Exponentially growing cells were transfected by electroporation (400 V, ∞ Ω, 125 μF, Bio-Rad Gene Electropulser II). About 10-μg of DNA was used per transfection (2 × 10^6^ cells). Cells were analysed 24–48 h after transfection. Human 5-HT3A-myc- and 5-HT3B-HA-tagged cDNAs were expressed from the mammalian expression vector PRK5JD (Boyd *et al*., 2002).

### Radioligand binding

[^3^H]granisetron binding was performed on intact transiently transfected COS-7 cells cultured in 24 or 96-well plates (Cheng *et al*., 2007). Mock-transfected cells were used to determine the background binding signal. Cells were incubated in serum-free media (Opti-MEM) ± test drugs as indicated and then washed twice with opti-MEM or as indicated. Cells were washed with ice-cold binding buffer (10 mM HEPES, 135 mM NaCl, 5 mM KCl, 1 mM CaCl_2_, 1 mM MgCl_2_, pH 7.4) and incubated in 3 nM [^3^H]granisetron (in binding buffer) for 120 min on ice. Excess radioligand was removed by two washes with ice-cold binding buffer. Cells were then solubilized with 2% TX-100, and the remaining radioactivity counted by placing in scintillation cocktail. Background (mock) binding was subtracted from the signal, which was expressed as a percentage of signals present in untreated cells.

In competition binding experiments, cells were incubated with 0.6 nM [^3^H]granisetron in the presence of a range of concentrations of inhibitor for 120 min at 19°C. Excess radioligand and counting was performed as above. Data were then plotted using one-site competition analysis in GraphPad Prism 4 (GraphPad Software, Inc., San Diego, CA, USA), and K_i_ values derived by the Cheng-Prusoff equation using a published K_d_ value of 0.55 nM for [^3^H]granisetron (Thompson *et al*., 2011).

### Cell surface ELISA

Methods were adapted from those published previously (Bollan *et al*., 2008). Transiently transfected COS-7 cells were grown on poly-L lysine coated 96-well plates. Cells were incubated in Opti-MEM ± test drug for 150 min at 37°C. All subsequent solutions were made in PBS (137 mM NaCl, 2.7 mM KCl, 10 mM Na_2_PO_4_, 1.8 mM KH_2_PO_4_, 1 mM CaCl_2_, 1 mM MgCl_2_, pH 7.4) unless stated otherwise. Following experimental treatment, cells were fixed in ice-cold 3% paraformaldehyde for 15 min and then washed with PBS. To reduce background signal, cells were incubated in 0.1 M glycine for 60 min, washed and then incubated in 3% H_2_O_2_ for 5 min, washed again and then blocked for 60 min in PBS with 5% FBS and 1% BSA (blocking solution). Primary antibody (in blocking solution) was incubated for 60 min and then washed three times with blocking solution. Secondary antibody (rabbit anti-sheep HRP diluted to 200 μg·mL^−1^ in block or sheep anti-mouse HRP diluted to 400 μg·mL^−1^ in blocking solution) was then incubated for 60 min and subsequently washed four times in PBS. Cells were then incubated with Amplex UltraRed (19 μM) and H_2_O_2_ (740 μM) for 15 min in the dark, and the plate read at excitation/emission wavelengths of 530/590 nm. Background (mock) readings were subtracted from the signal and values are expressed as percentage of untreated cells.

### Immunofluorescence antibody feeding experiments

Methods were adapted from those described previously (Connolly *et al*., 1999). Transiently transfected COS-7 cells were grown on poly-L lysine coated coverslips. Coverslips were washed in ice-cold PBS then incubated in 50 μg·mL^−1^ sheep anti-myc antibody or mouse anti-HA antibody (diluted in PBS) on ice for 60 min. Coverslips were then washed in PBS and incubated in Opti-MEM ± test drug for 40 min on ice (to allow pre-equilibration of drugs), before incubating at 37°C for 40 min to permit endocytosis. Surface-bound myc antibody was removed by acid stripping (PBS at pH 2.5) followed by neutralization (PBS at pH 7.4). Cells were fixed (3% paraformaldehyde, 15 min), permeabilized (0.5% TX-100 in PBS, 10 min), quenched twice (50 mM NH4Cl, 0.05% TX-100 in PBS), then blocked (10% FBS, 0.5% BSA, 0.05% TX-100 in PBS) for 60 min. Parallel sets of cells were not permeabilized in order to confirm removal of all surface receptor-bound antibody. Internalized receptors were detected with anti-sheep (Alexa Fluor 594) or anti-mouse (Alexa Fluor 568) antibody (10 μg·mL^−1^ in PBS, 30 min at room temperature) and finally washed four times in PBS. Cells were examined using a wide field imaging system and Volocity (Perkin Elmer) software.

### Data analysis

The data shown represent the mean ± SD of at least three experiments, performed with three (binding experiments) or eight (ELISA) replicates. Statistical differences between means were assessed using one-way ANOVA with Dunnett's *post hoc* test, or paired Student's t-test, as indicated. All data analysis was performed using GraphPad Prism 4.

### Materials

5-HT hydrochloride, ondansetron hydrochloride dihydrate, nystatin, dynasore hydrate, monensin sodium hydrate, Exo-1, cycloheximide, anisomycin and anti-HA/myc antibodies were from Sigma-Aldrich (Dorset, UK). Palonosetron hydrochloride was from Helsinn Birex (Dublin, Ireland). [^3^H]BRL-43964 ([^3^H]granisetron) was from PerkinElmer (Buckinghamshire, UK). Cell culture reagents, Amplex UltraRed, Alexa fluor 594 conjugated anti-sheep antibody and Alexa fluor 568 conjugated anti-mouse antibody were from Invitrogen (Paisley, UK). HRP conjugated anti-sheep and anti-mouse antibodies were from GE Healthcare (Buckinghamshire, UK).

## Results

### Palonosetron decreases cell surface 5-HT_3_ receptor binding sites

**Figure 1:**
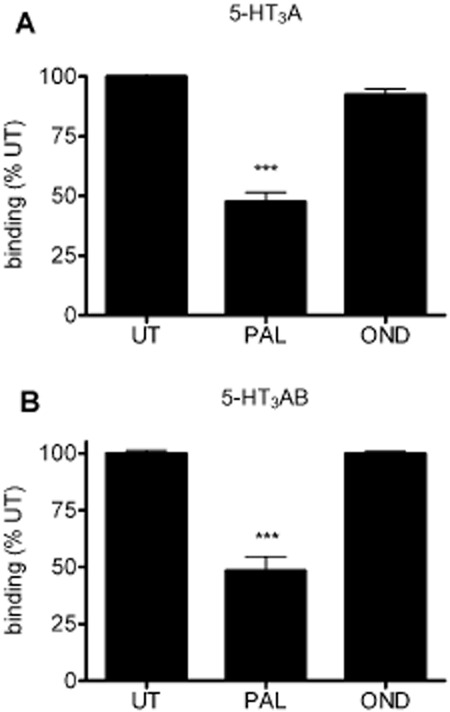
The long-term effect of prior exposure to 5-HT_3_ receptor antagonists on the availability of 5-HT_3_ receptor binding sites. [^3^H]granisetron binding was performed in COS-7 cells expressing 5-HT_3_A (A) or 5-HT_3_AB (B) receptors. Cells were incubated with palonosetron (PAL, 1 nM) or ondansetron (OND, 30 nM) for 150 min at 37°C and incubated in drug-free buffer for 150 min at 37°C with three washes. Remaining surface binding sites were detected using [^3^H]granisetron. Data are an average of at least three separate experiments performed in triplicate. ***P < 0.001, significantly different from untreated cells UT; anova.

**Figure 2:**
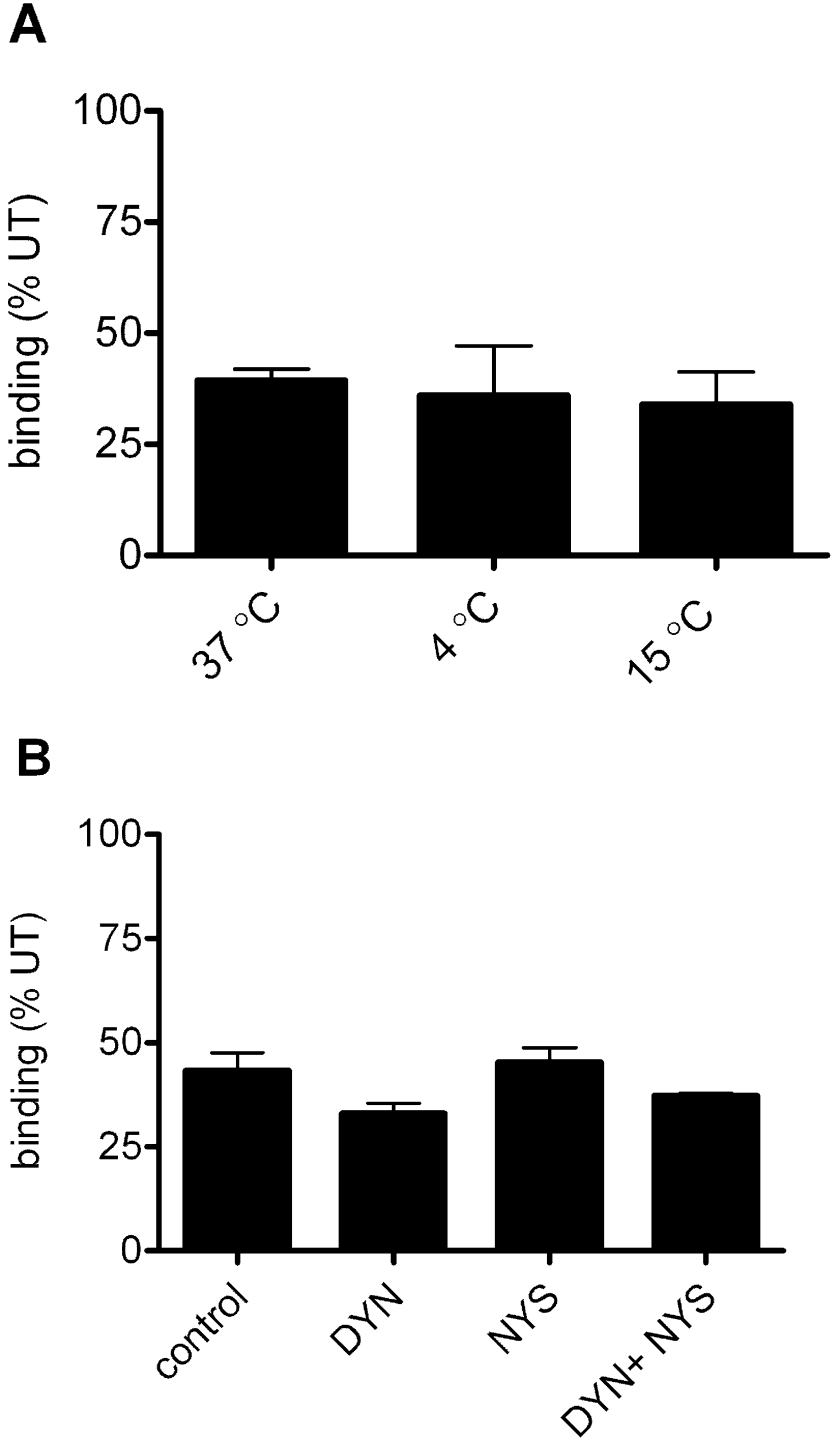
The inhibition of endocytosis does not prevent the palonosetron-mediated down-regulation of 5-HT_3_ receptor binding sites. [^3^H]granisetron binding was performed on 5-HT_3_A-expressing COS-7 cells. (A) Palonosetron (1 nM) pretreatment (150 min) at 4, 15 or 37°C and incubated in drug-free buffer for 150 min, at the same temperatures, prior to radioligand binding. (B) Palonosetron (1 nM) was incubated in the presence of dynasore (DYN, 80 μM), nystatin (NYS, 21 μM), or both and incubated at 37°C (150 min). Following incubation in drug-free buffer (150 min) radioligand binding was performed (37°C). Data are expressed as % of untreated cells and experiments were performed on three independent occasions in triplicate.

To investigate the effect of palonosetron on the availability of 5-HT_3_ receptor binding sites, [^3^H]granisetron was used to monitor available binding sites on the cell surface. In order to distinguish surface from intracellular receptors, we determined the level of [^3^H]granisetron binding to surface receptors by using low pH elution of surface bound ligand (see Supporting Information Figure S1). Under our conditions (no detergent present), [^3^H]granisetron bound exclusively to surface 5-HT_3_ receptors, with little or no binding to intracellular sites. To determine whether prior exposure to palonosetron causeds a long-term loss of 5-HT_3_ receptor binding sites, as reported previously (Rojas *et al*., 2008; 2010b) we incubated 5-HT_3_A receptor expressing cells with palonosetron (1 nM) for 150 min at 37°C followed by removal of unbound ligand and incubation in drug-free media for 150 min to permit palonosetron unbinding. The available surface ligand binding sites were determined with [^3^H]granisetron. In keeping with previous observations (Rojas *et al*., 2010b), palonosetron treatment significantly reduced the level of 5-HT_3_A receptor surface binding sites (*P* < 0.01; anova; *n* = 5) (Figure 1A). This could be an under-estimate if new receptor delivery to the cell surface occurs during the recovery period (at 37°C). However, no difference in recovery, compared with results from assays performed at 4°C or 15°C (Figure 2A), was evident, suggesting that no significant exocytosis of receptors occurred during this period. In contrast to the findings with palonosetron, ondansetron (30 nM) did not have any long-term effect on binding (Figure 1A).

To investigate the effect of 5-HT_3_ receptor antagonists on 5-HT_3_AB heteromeric receptors, cells were transfected with human 5-HT3A and 5-HT3B cDNAs at a ratio of 3:7 in order to favour expression of heteromers. As observed with 5-HT_3_A homomeric receptors, palonosetron, but not ondansetron, reduced the level of 5-HT_3_AB receptor binding (*P* < 0.01; anova; *n* = 3; Figure 1B).

### Palonosetron-induced down-regulation of 5-HT_3_ receptor binding sites is insensitive to the inhibition of endocytosis

As palonosetron-induced internalization of 5-HT_3_ receptors has been reported as a likely explanation for the observed long-term decreases in receptor binding (Rojas *et al*., 2010b), we sought to investigate whether the inhibition of endocytosis would prevent this down-regulation. The incubation of cells at low temperatures prevents internalization of receptors. However, incubation at either 4°C or 15°C, during palonosetron exposure (150 min) and a subsequent recovery period (150 min), did not alter the down-regulation of 5-HT_3_A receptor binding observed at 37°C (Figure 2A). Thus, palonosetron-induced down-regulation occurred at low temperatures where receptor internalization was not possible. To support this finding, we also investigated whether the down-regulation was independent of either clathrin- or caveolin-dependent endocytic pathways using the pharmacological inhibitors dynasore and nystatin, respectively (see Kiss, 2012). Co-incubation of palonosetron with either dynasore (80 μM), nystatin (21 μM) or both together, did not prevent the palonosetron-induced down-regulation of 5-HT_3_A receptor binding sites (Figure 2B). Taken together, these results indicate that the prolonged loss of 5-HT_3_ receptor binding sites did not result from receptor internalization.

### Palonosetron does not decrease cell surface 5-HT_3_ receptor populations

**Figure 3:**
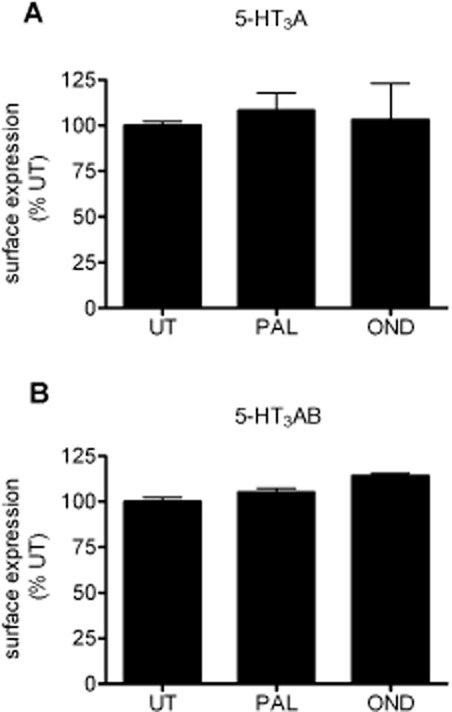
Cell surface levels of 5-HT_3_ receptors are not altered by palonosetron treatment. Cell surface ELISA was performed on COS-7 cells expressing 5-HT_3_A-myc (A) or 5-HT_3_A-myc/B-HA receptors (B), probing with anti-myc or –HA antibodies, respectively. Cells were incubated with palonosetron (PAL, 1 nM) or ondansetron (OND, 30 nM) for 150 min at 37°C and then fixed to prevent further membrane trafficking. Cell surface receptors were then probed using ELISA. Data are an average of three experiments performed in octuplicate and normalized to untreated (UT).

To confirm that the decrease in 5-HT_3_ receptor binding after exposure to palonosetron did not result from receptor internalization, we quantified cell surface receptor levels using cell surface ELISA (Bollan *et al*. 2003; Boyd *et al*., 2003). In cells expressing epitope-tagged 5-HT_3_ receptors (5-HT3A-myc and 5-HT3B-HA subunits), cell surface levels were determined for homomeric (5-HT_3_A) or heteromeric (5-HT_3_AB) receptors. As this approach targets the receptor epitope tag rather than the ligand binding site, it reports directly on surface receptor levels, independent of ligand binding and receptor inhibition by palonosetron. Following palonosetron (1 nM) or ondansetron (30 nM) binding (150 min, 37°C), the level of cell surface expression of either 5-HT_3_A (Figure 3A) and 5-HT_3_AB (Figure 3B) receptors was unchanged, compared with the untreated control.

**Figure 4:**
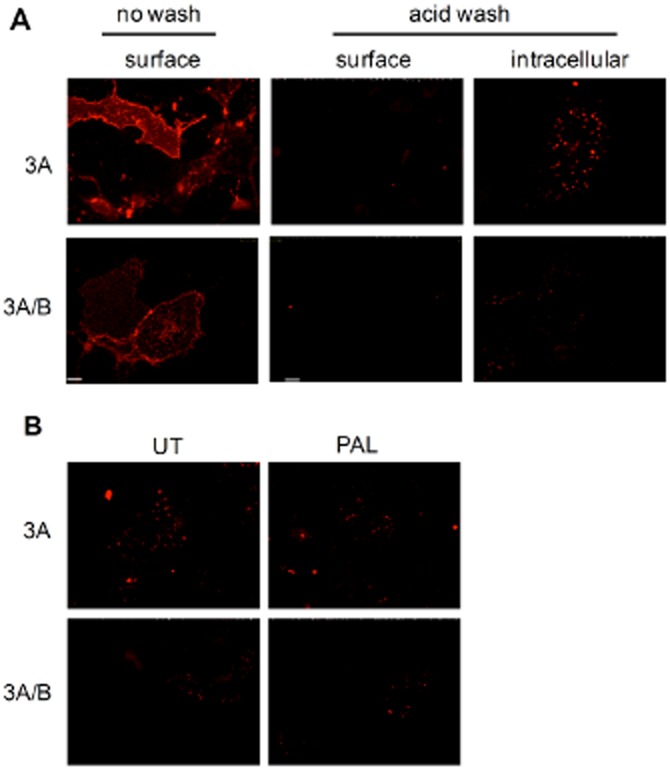
5-HT_3_ receptor internalization is not induced by palonosetron (PAL). (A) COS-7 cells expressing 5-HT_3_A-myc (3A) or 5-HT_3_A-myc/B-HA (3A/B) were probed with anti-myc or –HA antibodies (4°C, 60 min). Cell surface receptors were identified using Alexa 568 conjugated secondary antibody (surface). Receptor internalization (37°C, 30 min) was identified following the removal of surface antibody by a low pH (acid wash) and probed with the secondary antibody (surface). Internalized receptors were detected following permeabilization (intracellular). (B) Cells were pre-bound with primary antibodies, as above. They were then incubated in the absence or presence of palonosetron (1 nM, 30 min, 37°C). Cells were then permeabilized and probed with secondary antibody. Images represent at least three separate experiments. Scale bar = 50 μm. UT, untreated.

Although these results indicated that palonosetron (and ondansetron) did not down-regulate cell surface receptors, they did not comment on the rate of receptor internalization as receptors could be recycled or replaced by new receptors during the course of the experiment. Indeed, it has been reported that 5-HT_3_ receptors recycle constitutively, regardless of the absence (Morton *et al*., 2011) or presence (Ilegems *et al*., 2004; Freeman *et al*., 2006) of ligands. To address 5-HT_3_ receptor internalization, both constitutive and ligand-induced, we performed antibody-feeding experiments and fluorescence microscopy. The cells expressing 5-HT_3_A-myc or 5-HT_3_A-myc/B-HA receptors were labelled with anti-myc/HA antibodies (4°C, 60 min to prevent receptor internalization during labelling). Following labelling, cells were warmed to 37°C (30 min) to permit constitutive (no ligand present) internalization. Antibody remaining bound to cell surface receptors was removed by acid stripping. Internalized receptors were then detected using Alexa Fluor 568 conjugated secondary antibodies after permeabilization of the cells. To ensure the efficacy of the acid wash step to remove surface receptor-bound antibody, we probed with secondary antibody without permeabilization (Figure 4A, middle panels). Using this approach, low-level constitutive internalization (as indicated by the punctate staining pattern) was apparent for both 5-HT_3_A and 5-HT_3_AB receptors (Figure 4A, right panels). The inhibition of membrane trafficking by incubating at 4°C completely prevented the detection of intracellular receptors (not shown). In the presence of palonosetron (1 nM), no enhancement of the constitutive rate of internalization was evident (Figure 4B). In summary, palonosetron binding did not induce the internalization or down-regulation of the expression of either 5-HT_3_A or 5-HT_3_AB receptors.

### Recovery from palonosetron-mediated inhibition of 5-HT3 receptors suggests its slow dissociation from the receptors

**Figure 5:**
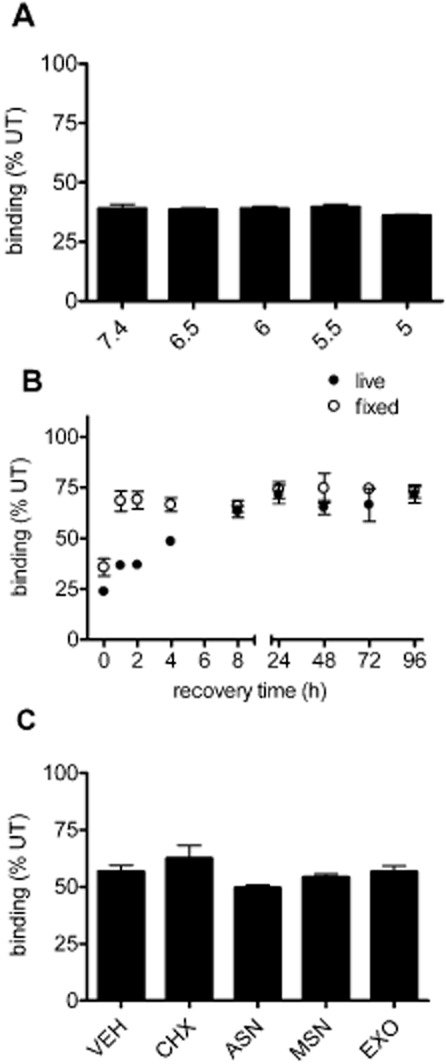
Recovery of 5-HT_3_ receptor binding sites following palonosetron exposure. (A) Cells were incubated with palonosetron (1 nM) for 150 min at 4°C, and then washed in acidic buffer (at indicated pH), prior to [^3^H]granisetron binding (performed at pH 7.4). (B) Following palonosetron exposure (1 nM, 150 min, 37°C) in either live or fixed cells, recovery of 5-HT binding sites was monitored over time (0–96 h) using [^3^H]granisetron binding and related to the level of binding sites in untreated (UT) cells. (C) Cells were allowed to recover for 8 h after removal of palonosetron in the absence (0.1% ethanol vehicle, VEH) or presence of cycloheximide (CHX, 35 μM), anisomycin (ASN, 10 μM), monensin (MSN, 5 μM) or Exo-1 (EXO, 50 μM). Radioligand binding signal is expressed as a % of control cells that were treated with inhibitor or vehicle but not exposed to palonosetron. Data are average of three independent experiments performed in triplicate.

Constitutively, internalized receptors (Figure 4) bound by palonosetron could unbind within the acidic endosomal environment and recycle back to the cell surface making them functionally available again. Therefore, we investigated whether palonosetron binding could be reversed under such acidic conditions. To investigate the pH sensitivity of the palonosetron-receptor interaction, we investigated whether palonosetron could be eluted from surface receptors using low pH. The 5-HT_3_A expressing cells were treated with palonosetron (1 nM, 4°C, 150 min) and washed (on ice) in binding buffer at a range of pH values (pH 7.4, 6.5, 6.0, 5.5 or 5.0), then incubated for 10 min (on ice) in buffer at each pH. The subsequent detection of 5-HT_3_ receptor binding sites (at pH 7.4), compared with untreated (no palonosetron) cells, revealed that palonosetron down-regulation could not be reversed by ligand elution at any pH examined and reduced [^3^H]granisetron binding following palonosetron exposure was still evident (Figure 5A). The untreated cells could still bind ligand following the low pH treatment (binding in cells pre-exposed to pH 5.0 was 112.3 ± 6.8% of cells exposed to pH 7.4), suggesting that receptor binding capacity was not irreversibly affected by the low pH conditions. Therefore, the palonosetron-receptor interaction was not sensitive to the low pH encountered within endosomes. Thus, any recycled receptor undergoing constitutive internalization during palonosetron exposure would remain inhibited.

To investigate the time-course of recovery from down-regulation by palonosetron, 5-HT_3_A expressing cells were treated with palonosetron (1 nM, 150 min, 37°C) and excess ligand removed. Cells were then allowed to recover in culture media for 0, 1, 2, 4, 8, 24, 48, 72 or 96 h (37°C) and cell surface receptors levels were measured with [^3^H]granisetron. The inhibitory effect of palonosetron on 5-HT_3_ receptor binding were still evident 96 h after its removal, with binding at significantly reduced levels compared to untreated time-matched control (*P* < 0.05 paired *t*-test *n* = 3) at 71.5 ± 7.0%. Nevertheless, 5-HT_3_ receptor binding levels do exhibit some recovery between 0 and 8 h after its removal, after which time a plateau was reached. The rate of recovery demonstrated a t_1/2_ of 4.1 h.

As recombinant expression results in robust constitutive expression, recovery could be maximal due to ongoing receptor synthesis and transport to the cell surface. To investigate the mechanism by which 5-HT_3_ receptor binding recovery occurs, we used inhibitors of protein synthesis (35 μM cycloheximide or 10 μM anisomycin) or inhibitors of protein exit from the endoplasmic reticulum (5 μM monensin or 50 μM Exo-1); as demonstrated previously (Feng *et al*., 2003). Cells were incubated with palonosetron (1 nM, 150 min, 37°C), washed and then allowed to recover for 8 h (37°C) in the presence of each inhibitor or vehicle control (0.1% ethanol), before measuring the binding sites (Figure 5C). After 8 h, recovery from palonosetron was evident under normal conditions (Figure 5B). As constitutive internalization, seen previously (Ilegems *et al*., 2004; Freeman *et al*., 2006) and in this study (Figure 4), may indicate a requirement for the continual turnover of 5-HT_3_ receptors, we accounted for this in our study by including each inhibitor in the control (no palonosetron) condition, and binding in palonosetron-treated cells was expressed as a percentage of each of these. In all cases, recovery of 5-HT_3_ receptor binding sites still occurred (Figure 5C; *P* > 0.05; anova; *n* = 3). Thus, this data suggests that recovery from palonosetron treatment does not require the delivery of new receptors to the cell surface.

As an alternative approach to investigate the role of 5-HT_3_ receptor delivery to the recovery of binding sites after exposure to palonosetron, cells were fixed (3% paraformaldehyde, 15 min) to completely prevent membrane trafficking and create a static population of receptors with which to investigate the time-dependency of recovery. In fixed cells, recovery of receptor binding sites still occurred (Figure 5B), providing further evidence that delivery of new receptors was not required for the time-dependent increase in binding sites observed in live cells. These findings suggested that the recovery of binding sites occurred as a result of dissociation of palonosetron from the receptors. The rate of recovery observed in fixed cells was quicker than in live cells, which may reflect subtle differences in the receptor binding site or recovery following receptor conformational changes and/or receptor recycling. Recovery in both fixed and live cells was not complete, reaching a plateau of approximately 75% (of untreated) that was maintained for at least 96 h. These findings indicate an additional irreversible binding mode of palonosetron that is distinct from the slowly dissociating binding, and may reflect multiple sites of interaction of palonosetron with 5-HT_3_ receptors.

### The potency of palonosetron-mediated down-regulation of 5-HT_3_ receptors is closely matched to its receptor binding affinity

**Figure 6:**
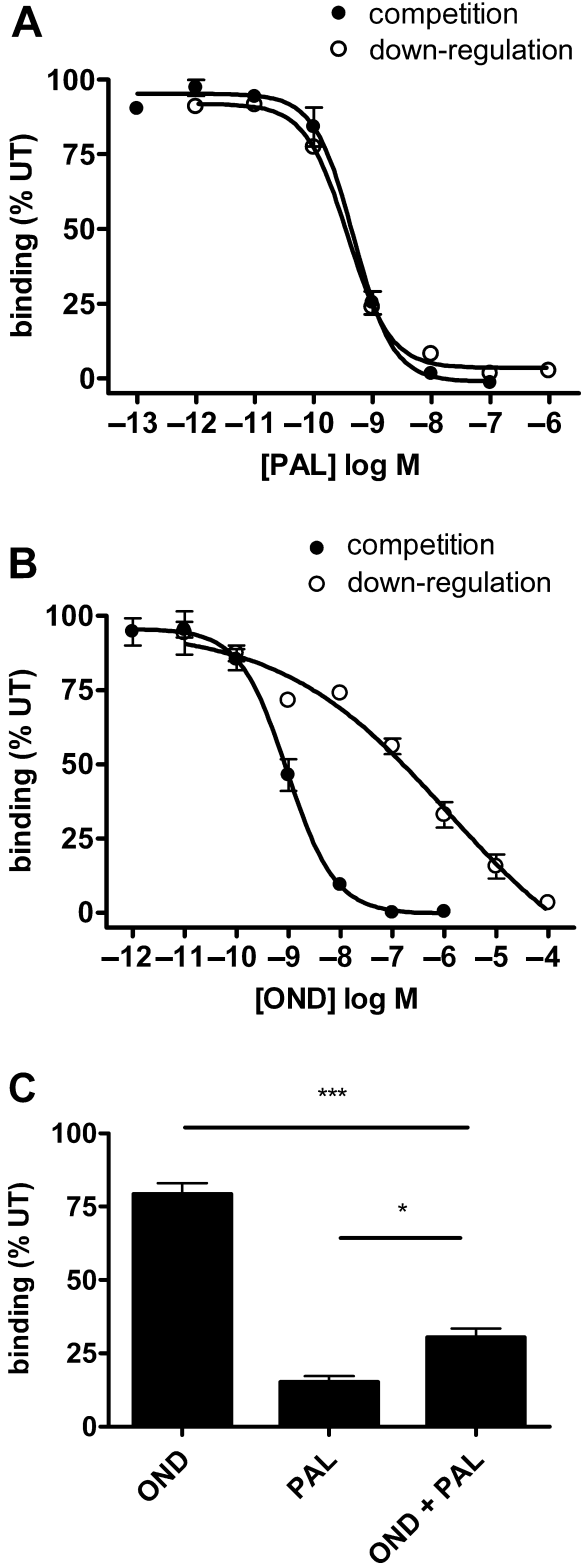
Concentration-dependency of 5-HT_3_ receptor binding and down-regulation by palonosetron and ondansetron. A range of concentrations of palonosetron (A) and ondansetron (B) were tested for their ability to compete for [^3^H]granisetron binding (competition binding) or cause chronic 5-HT_3_ receptor down-regulation. Competition binding was performed by co-incubating 5-HT_3A_ receptor expressing cells with palonosetron (PAL) or ondansetron (OND) with 0.6 nM [^3^H]granisetron for 120 min at 19°C. Down-regulation was measured by incubating cells with palonosetron or ondansetron for 150 min at 19°C, washing excess ligand and then incubating in drug-free media for a further 150 min at 19°C before binding to [^3^H]granisetron. (C) Cells were incubated with or without 10 nM ondansetron for 30 min at 19°C, followed by incubation with 1 nM palonosetron plus 10 nM ondansetron, or 10 nM ondansetron alone for 150 min at 19°C. [^3^H]granisetron binding was performed after washout of excess ligand and incubation in drug-free media for 150 min at 19°C. Binding was expressed as a percentage of untreated (UT) cells. Data represent an average of three experiments performed in triplicate. *P < 0.5, ***P < 0.001, significantly different as shown; anova.

We next examined the relationship between the binding affinity of palonosetron and the concentration-dependency of its ability to chronically down-regulate 5-HT_3_ receptors. To measured binding affinity, competition binding was performed with 0.6 nM [^3^H]granisetron (close to K_d_ for this ligand) for 120 min. In these experiments, binding was performed at 19°C to inhibit receptor endocytosis. Palonosetron exhibited a K_i_ value of 0.22 ± 0.07 nM (Figure 6A).

To determine the concentration-dependent relationship of palonosetron-mediated 5-HT_3_ receptor down-regulation, cells were exposed to a range of concentrations of palonosetron for 150 min at 19°C, excess ligand washed out, and the cells incubated in drug-free media for a further 150 min at 19°C before [^3^H]granisetron binding was performed at 4°C. Palonosetron exhibited a concentration-dependent decrease in binding sites with an IC_50_ of 0.38 ± 0.02 nM (Figure 6A), indicating that down-regulation occurs within a concentration range very similar to that of competition binding (dose ratio of 1.7 between the two measurements).

Similar experiments were conducted with ondansetron (Figure 6B). Competition binding studies demonstrated that ondansetron also has high 5-HT_3_ receptor binding affinity, with a K_i_ value of 0.47 ± 0.14 nM. However, when tested for its ability to down-regulate receptors, ondansetron caused chronic inhibition of binding at high concentrations. This down-regulating effect of ondansetron had an IC_50_ value of 907 ± 84.9 nM. Therefore, in the case of ondansetron, receptor binding and receptor down-regulation occur at clearly different concentration ranges (dose ratio of 1929.8), consistent with the possibility that ondansetron has different affinities for distinct receptor binding sites.

### Palonosetron-mediated down-regulation of 5-HT_3_ receptors may involve allosteric receptor interactions

In order to further probe the mechanisms by which receptor down-regulation occurs, we took advantage of the different concentration-dependencies of palonosetron and ondansetron for producing acute and chronic receptor inhibition (competition and down-regulation, respectively, Figure 6A,B). By the co-application of 10 nM ondansetron, we should selectively block palonosetron binding to the competitive binding site and inhibit down-regulation if this results from binding to the same site. However, if down-regulation occurs via binding to an allosteric binding site, down-regulation would be unaffected. To test this, cells were pre-incubated with or without 10 nM ondansetron for 30 min before exposure to 1 nM palonosetron for 150 min (with or without the continued presence of 10 nM ondansetron). Excess ligand was washed out and the cells incubated for a further 150 min in drug-free media. All incubation and wash stages were performed at 19°C.

At 10 nM, ondansetron alone resulted in a small down-regulation compared with that of 1 nM palonosetron (Figure 6C). In the presence of ondansetron, palonosetron still caused robust chronic down-regulation of receptor binding sites but to a lesser extent. Therefore, ondansetron caused a partial blockade of palonosetron-mediated chronic receptor down-regulation, consistent with a role of allosteric receptor interactions of palonosetron in causing this effect.

## Discussion

Palonosetron represents an important treatment for emesis and nausea caused by cancer therapy (chemotherapy or radiotherapy) or surgical procedures, and a growing body of evidence suggests that it is the most effective of the 5-HT_3_ receptor antagonists in these settings, exhibiting efficacy in both acute and delayed cases (Aapro, 2007; Muchatuta and Paech, 2009; Rojas and Slusher, 2012; Schwartzberg *et al*., 2012).

Using radioligand binding, we confirmed previous studies showing that palonosetron caused long-term (several hours) inhibition of cell surface 5-HT_3_A receptor binding sites (Rojas *et al*., 2010a,b; Rojas and Slusher, 2012), and extended these to include 5-HT_3_AB heteromeric receptors. We also demonstrated a longer term inhibition of the receptors (several days) by palonosetron and this effect was shared by ondansetron at higher concentrations. As palonosetron competes for the same binding site as the radioligand ([^3^H]granisetron) (Eglen *et al*., 1995; Wong *et al*., 1995; Rojas *et al*., 2008; Moura Barbosa *et al*., 2010) these findings could have one of two explanations. Either palonosetron promotes internalization of 5-HT_3_ receptors and hence a reduction in cell surface binding sites, or it has prolonged activity at the receptor, due to slow dissociation kinetics or irreversible binding. A series of experiments were conducted to distinguish between these mechanisms. By blocking pathways of cellular endocytosis, we showed that down-regulation did not require 5-HT_3_ receptor internalization. These findings were further corroborated by cell surface ELISA, which demonstrated that the number of cell surface 5-HT_3_ receptors was unchanged after chronic exposure to palonosetron. Despite the existence of constitutive 5-HT_3_ receptor internalization (Morton *et al*., 2011), this was not enhanced by exposure to palonosetron.

Taken together, these results suggest that the prolonged inhibitory effects of palonosetron occur in the presence of long-lasting cell surface receptors. Therefore, palonosetron may act as an irreversible antagonist, forming permanent interactions with human 5-HT_3_ receptors. Alternatively, it might be a pseudo-irreversible antagonist, where very slow dissociation kinetics effectively causes permanent antagonism over the time-course of a normal experiment (Kenakin, 1997). To investigate this further, time-course experiments examining the recovery of 5-HT_3_ receptor binding sites from palonosetron were conducted. Recovery was incomplete and slow (t_1/2_∼4 h), requiring around 8 h before the appearance of 5-HT_3_ receptor binding sites plateaued. In this study, the recovery that did occur did not require new receptor delivery, suggesting a slow dissociation of palonosetron. If the prolonged inhibitory effects of palonosetron are truly irreversible (beyond 4 days), recovery will require the delivery of new receptors to the plasma membrane. That palonosetron has two binding modes (one slowly dissociating and one persistent) is consistent with the possibility of two distinct binding sites. Indeed, palonosetron binds allosterically to 5-HT_3_ receptors (Rojas *et al*., 2008; Moura Barbosa *et al*., 2010), and such interactions may influence subsequent inhibition of [^3^H]granisetron binding.

Further evidence of multiple 5-HT_3_ receptor binding sites is suggested by the inability of excess ondansetron (as determined by competition binding) to completely block palonosetron-induced down-regulation. However, at higher concentrations, ondansetron was also capable of causing receptor down-regulation. This may indicate the existence of an orthosteric site for ondansetron at which competition binding occurs (<1 nM affinity) and a putative allosteric site at which down-regulation occurs (∼1 μM affinity). In contrast, palonosetron would have high affinity for both these sites, in the <1 nM range, as demonstrated by its similar concentration-dependency for competition and down-regulation.

That palonosetron binding is robust and pH-insensitive was also consistent with binding to an atypical alternative 5-HT_3_ receptor site, as previously described (Eglen *et al*., 1995; Wong *et al*., 1995; Rojas *et al*., 2008; Moura Barbosa *et al*., 2010). Importantly, this pH-insensitivity highlights the fact that even receptors internalized into cells by constitutive endocytosis would not release their bound palonosetron within the acidic endosomal pathway (early endosomes, pH 6.5, multi-vesicular bodies, pH 5.5 and late endosomes/lysosomes, pH 5.0). Furthermore, palonosetron is an insurmountable 5-HT_3_ receptor antagonist as it prevents 5-HT from evoking maximal responses (Wong *et al*., 1995). This is indicative of prolonged receptor inhibition, which effectively removes receptors from the total pool on which an agonist can act, rather than the formation of a simple association and dissociation equilibrium between the antagonist and agonist. In previous studies, 5-HT_3_ receptor antagonists possessing a quinuclidine ring (which includes palonosetron, but not ondansetron) irreversibly inhibit the receptors through the putative formation of covalent bonds (Langlois *et al*., 1994). This is further supported by the fact that kinetic binding studies have demonstrated that radiolabelled palonosetron remains associated with cells expressing 5-HT_3_ receptors for hours (Rojas *et al*., 2010b). However, these authors also found that palonosetron dissociates completely (within an hour) in isolated membranes and attribute this difference to an intracellular localization of palonosetron in intact cells due to internalization. Indeed, they reported that palonosetron decreased cell surface populations of fluorescently tagged murine 5-HT_3_ receptors (Rojas *et al*., 2010b). Such discrepancies between this and the present study may reflect differences between mouse (Rojas *et al*., 2010b) and human (this study) 5-HT_3_ receptors. For example, it is known that the effects of the chaperone protein RIC-3 are different in mouse and human, with the abolition or enhancement of receptor expression in mouse or human, respectively (Halevi *et al*., 2003; Cheng *et al*., 2007). An alternative explanation for the relatively rapid and complete dissociation of palonosetron binding in cell membranes could be that putative alternative binding sites on 5-HT_3_ receptors (that cause prolonged inhibition) are not present in the same form, as in intact living cells. Additionally, as direct evidence for internalization was observed at only very low levels with a fluorescently tagged 5-HT_3_ receptor construct (Rojas *et al*., 2010b), the large tag insertion may disrupt the presentation of internalization signals within the receptor causing subtle changes in receptor behaviour. Furthermore, the internalized receptors reported by these authors lack the punctate staining pattern typical of endosomal localization as observed here and by others (Morton *et al*., 2011) but instead appear more consistent with labelling of endoplasmic reticulum-resident receptors in the biosynthetic pathway. Nevertheless, in the present study, ELISA experiments demonstrated no observable change in surface receptor populations upon exposure to palonosetron when taking an average response of hundreds of thousands of cells, compared to only 12 cells in the earlier study.

The prolonged activity of palonosetron at the level of 5-HT_3_ receptor binding that we report here relates well to findings that it has long-lasting *in vivo* antiemetic activity in animal models (Eglen *et al*., 1995) and with its clinical efficacy in the treatment of delayed emesis (Rojas and Slusher, 2012; Schwartzberg *et al*., 2012). Importantly, the persistent effects of palonosetron we have demonstrated occur within the concentration range (nM) of this drug found in the blood for days after administration of the recommended dosage (Eisenberg *et al*., 2004). Interestingly, the peak clinical plasma concentration of ondansetron is higher, at approximately 0.1 μM (Moreira *et al*., 2010; Musshoff *et al*., 2010), which is more consistent with a clinical requirement to target its efficacy in driving chronic down-regulation, rather than its receptor affinity. This raises the question as to whether chronic receptor inhibition is a prerequisite for the clinical efficacy of 5-HT_3_ antagonists in general. Perhaps, the improved clinical efficacy of palonosetron is related to its high potency in receptor down-regulation, allowing chronic down-regulation to occur at lower and more clinically attainable plasma concentrations. Clinically relevant plasma levels over time after administration for palonosetron have been shown to be approximately 3 nM (1–2 h), 1.5 nM (24 h) and 0.3 nM (7 days) (Eisenberg *et al*., 2004) that should lead to down-regulation of 90%, 75% and 50% respectively. For ondansetron, plasma levels are 0.1 μM (1–2 h) and 0.5 nM (24 h hours) (Moreira *et al*., 2010) that could achieve down-regulation by 50 and 20%, respectively. In addition to its long duration in the body (t_1/2_ > 40 h) (Eisenberg *et al*., 2004), the effects of palonosetron might persist well beyond its clearance or metabolism due to its potency in driving receptor down-regulation. Indeed, palonosetron has been found to be effective in preventing emesis during chemotherapy for up to 5 days after treatment (Eisenberg *et al*., 2003; Gralla *et al*., 2003; Aapro *et al*., 2006).

Clearly, the role of 5-HT_3_ receptors is important in normal physiology (Hansen and Witte, 2008; Liu *et al*., 2011) and in irritable bowel syndrome (Spiller, 2008; Niesler, 2011) and their permanent inhibition would be undesirable. Perhaps palonosetron achieves the optimal period of time for 5-HT_3_ receptor inhibition that prevents delayed emesis, but has consequences tolerable to normal physiological functions.

## Abbreviations

RIC-3: resistance to inhibitors of cholinesterase type-3

## Supporting information

**Figure S1** Acid wash conditions to elute surface receptor binding of [^3^H] granisetron. [^3^H] granisetron binding in COS-7 cells transfected with human 5-HT_3_A myc was eluted by a wash in neutral (pH 7.4) or acidic (pH 2.5) PBS. Eluted counts (black bar) and cell-retained counts (grey bar) were quantified by Scintillation counting. Following a neutral wash, most counts (98.2 ± 3.7%) were associated with the cells. In contrast, the acidic wash eluted all counts (100 ± 7.0%). Therefore, all [^3^H] granisetron binding sites are present at the cell surface in these experiments and are completely sensitive to low pH. Data represent an average of three experiments, each performed in triplicate.
